# Repetitive Transcranial Magnetic Stimulation (rTMS) for the cognitive rehabilitation of traumatic brain injury (TBI) victims: study protocol for a randomized controlled trial

**DOI:** 10.1186/s13063-015-0944-2

**Published:** 2015-10-05

**Authors:** Iuri Santana Neville, Cintya Yukie Hayashi, Simone Alves El Hajj, Ana Luiza Costa Zaninotto, Juliana Perez Sabino, Leonardo Moura Sousa, Marcia Mitie Nagumo, André Russowsky Brunoni, Barbara Dal Forno Silva Shieh, Robson Luis Oliveira Amorim, Manoel Jacobsen Teixeira, Wellingson Silva Paiva

**Affiliations:** Division of Neurosurgery, Hospital das Clínicas, University of São Paulo, Rua Dr Eneas Aguiar, 255 / 4079; 05403-010, São Paulo, SP Brazil; Service of Interdisciplinary Neuromodulation, University of São Paulo, São Paulo, Brazil; Nursing School, University of São Paulo, São Paulo, Brazil; Division of Neuropsychology, Hospital das Clínicas, University of São Paulo, São Paulo, Brazil; Institute of Psychiatry, Hospital das Clínicas, University of São Paulo, São Paulo, Brazil

**Keywords:** Brain Injury, Cognition, Cognitive, Diffuse Axonal Injury, Non-invasive brain stimulation, Psychology, Repetitive transcranial magnetic stimulation (TMS, rTMS)

## Abstract

**Background:**

Repetitive Transcranial Magnetic Stimulation (rTMS) has been proposed as a new tool in neurological rehabilitation of victims of traumatic brain injury (TBI). However, its usefulness to treat this condition has never been tested rigorously. The primary goal is to conduct a study protocol to determine whether rTMS used to cognitive rehabilitation of victims of TBI with diffuse axonal injury (DAI) is a safe instrument and if it enhances cognitive function recovery.

**Methods:**

Double-blind randomized controlled trial of patients with diffuse axonal injury. Thirty-six patients will be randomized to either an active coil group or sham group in a 1:1 ratio. rTMS protocol: 10 sessions of high-frequency rTMS (10 Hz) over the left dorsolateral prefrontal cortex (DLPFC). Cortical Excitability measures will be obtained. Neuropsychological evaluations will be performed 1 week before, 1 week and 3 months after rTMS. There are 2 study hypotheses: (1) rTMS over the left DLPFC in patients with DAI will improve cognitive function and (2) whether rTMS is safe in TBI patients.

**Discussion:**

This study evaluates the immediate and delayed effects of rTMS over the DLPFC on the cognitive domain of patients with DAI following TBI. rTMS has shown good results in treating major depression and may be promising for patients with TBI. As such, the results of this study can greatly modify the cognitive rehabilitation strategies.

**Trial registration:**

This trial was registered in clinicaltrials.gov (NCT02167971) on 17 June 2014.

## Background

### Epidemiology

Traumatic brain injury (TBI) remains as a global health problem that generates a major socioeconomic impact worldwide. Estimates indicate that TBI accounts for 9 % of global mortality and, more significantly, each death is related to dozens of hospitalizations, hundreds of medical appointments at the emergency departments and thousands of ambulatory consultations [1, 2]. This significant global adverse impact of TBI includes a major impact on Brazil, since cognitive sequelae among the survivors increase each year. According to Maset et al. [3], 360 cases/100,000 inhabitants per year is the reported incidence of TBI in Brazil.

### Pathophysiology

Traumatic axonal injury, also referred as diffuse axonal injury (DAI), is responsible for almost one third of deaths due to TBI and is the leading cause of disability among survivors, including not only motor deficits but also cognitive impairment and mood disorders [4, 5].

Regarding the pathogenesis of DAI, the basic structural damage is axotomy. Two basic mechanisms are suggested: primary axotomy and secondary axotomy. Primary axotomy occurs immediately after the tissue injury, allowing calcium influx and activation of the inflammatory cascade. Then follows secondary axotomy which happens hours after the trauma and can last for many years [6–9].

### Neuropsychological aspects

TBI is associated with a variety of disturbances in cognition and transient neurological deficits [10]. DAI is a common mechanism of injury in brain trauma associated with cognitive impairment, emotional and behavior disorders [11]. Cognitive impairment can be persistent, especially in moderate and severe injuries and deficits include: decreased executive functions and attentional process, judgment, verbal fluency, information processing and memory [12–14]. DAI patients can show diffuse cognitive impairments that may change over time due to secondary injury sequelae and Wallerian degeneration [15]. Imaging studies identified that the brain regions typically involved in DAI are: frontal regions, cingulate, thalamus and corpus callosum, resulting in decreasing of global intelligence [16], losses in executive functions, episodic memory and attentional process [17, 18].

### Rationale for a neuromodulation study in TBI

The number and type of neuropsychiatric conditions being treated by repetitive Transcranial Magnetic Stimulation (rTMS) is ever increasing [19, 20].

Several studies have demonstrated improvement in some cognitive aspects after rTMS [20–24]. Notably, rTMS may have cognitive enhancing properties and is under investigation for a variety of drug-resistant disorders [20]. A substantial and growing interest for the rTMS application of TMS in TBI patients has been seen, with encouraging results for the treatment of specific symptoms [25–28].

The rational use of rTMS for DAI patients is based on the perspective that induction changes in cortical excitability may lead to reorganization of a network responsible for an impaired cognitive function. This function may be restored or compensated by mechanisms involving structural and functional changes in brain circuits [25–27].

The intended use of rTMS is to enhance positive neuroplastic changes that could represent real improvement in the cognitive domain (such as working memory), quality of life and in mood disorder. Thus, neuropsychological assessments on rTMS studies can help measuring early and late cognitive and behavioral changes on TBI patients after the intervention.

### Study purpose and objectives

The clinical goal is to assess safety and determine whether high-frequency rTMS of the left dorsolateral prefrontal cortex (DLPFC) is effective for the cognitive rehabilitation of DAI patients after TBI.

### Primary outcome measures

The primary hypothesis is that the active coil group will show improvement between baseline and last early assessment of > 1 standard deviation on the Trail Making Test part B (TMTB), in comparison to the sham-treated patients The TMTB is known for its accurate assessment of executive function in mild and moderate TBI.

### Secondary outcome measures

- Evaluate the complications and technical difficulties of the proposed protocol
- Assess early and late performance after rTMS on the neuropsychological battery, which will include: memory and executive functions, attention, learning and processing speed; intensity of depressive symptoms, and intensity of anxious symptoms
- Compare cortical excitability with single-pulse and paired-pulse TMS between the active and sham coil groups.

## Methods

### Protocol proposal

#### General aspects

By the time this protocol was written in 2014, there were over 10,000 TMS manuscripts cataloged on PubMed [29], with at least 2,000 focusing on rTMS. However, to the best of our knowledge, there is no randomized controlled trial with rTMS in patients with TBI. Therefore, we propose the application of a promising instrument, which has already proved to be effective in many psychiatric and neurological conditions, in a population of chronic subjects, many of them disabled, with very few treatment options in terms of cognitive functional recovery.

#### Study design

This is a prospective, single-center, randomized, parallel-group controlled trial that will be held at the Hospital das Clínicas, University of São Paulo, São Paulo, Brazil. Study Recruitment started in January 2014 and the estimated study completion date for the primary outcome is September 2015.

This trial will follow the main CONSORT (Consolidated Standards of Reporting Trials) guidelines as well as its extension to non-pharmacological interventions. This protocol will also adhere to SPIRIT (Standard Protocol Items: Recommendations for Interventional Trials) guidelines.

#### Inclusion criteria

Patients older than 18 and younger than 60 years will be included. Only patients with clinical and radiological diagnosis of traumatic DAI after 1 year of TBI will be included. All the patients have to be able to answer the neuropsychological battery.

#### Exclusion criteria

Patients will be excluded if they have any of the following:

1. Prior history of epilepsy
2. Extensive cranial vault defects
3. Drug addiction
4. Pregnancy or are lactating mothers
5. Have a cardiac pacemaker or a cochlear implant
6. Have an implanted device (e.g. deep brain stimulator, ventriculoperitoneal shunt) or metal in the brain (e.g. aneurysm clip)
7. Known psychotic disorder
8. TBI within last 12 months

### Study intervention

#### Standard care

All patients will keep their clinical follow-up at the Neurotrauma Outpatient Clinics independently of the study and group assignment by other physicians not involved in this study.

### rTMS protocol

#### Magnetic stimulator

The rTMS will be performed with a commercially available MagPROX100 (Magventure Tonika Elektronic, Farum, Denmark) equipped with a focal figure-of-8-shaped coil (active coil model: MCF-B65 and sham coil model: MCF-P-B65) with continuous water cooling system to prevent overheating during stimulations. The sham coil has the same size and shape of the active coil. It also emits a noise very similar to the active one, but it does not create any significant magnetic field.

#### Target

rTMS will be applied with a figure-of-8-shaped coil centered over the left DLPFC, based on the F3 position of the International 10–20 system, using the modification described by Beam et al. [30]. We will perform 10 daily sessions in both groups (5 consecutive weekdays, 2 days-off during weekends and another 5 consecutive weekdays).

#### rTMS sessions

Every session will be held at the Service of Interdisciplinary Neuromodulation, *Hospital das Clínicas* – University of São Paulo, with the same machine and at the same daytime. Each session will consist of 50 trains of 40 pulses on each train separated by 25-second pauses applied at 10 Hz frequency, at an intensity of 110 % of the patient’s resting motor threshold (RMT) intensity.

### Resting motor threshold

RMT intensity was defined as the lowest stimulation intensity that, in 10 trials, induced at least 5 motor evoked potentials of at least 50-μV peak-to-peak amplitude assessed on the first dorsal interosseus muscle in the resting state.

#### Cortical excitability

The measurements will be performed before rTMS sessions, immediately after the last rTMS session (10th session) and 90 days after the completion of stimulations and will include: RMT measured in percentage of the maximum machine output, short-interval cortical inhibition (SICI) with interstimulus interval (ISI) of 2 and 4 mS, and intracortical facilitation (ICF) with ISI of 10 and 15 mS, both of them measured by paired-pulse TMS.

**Fig. 1:**
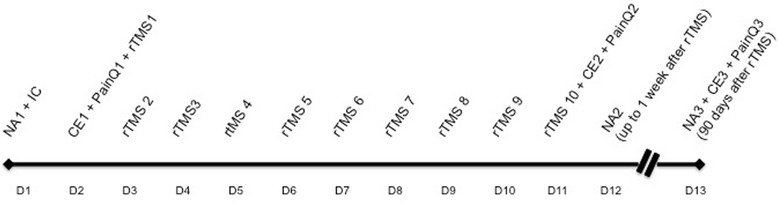
Timeline: this figure summarizes the study protocol. There will be 13encounters (D1–D13): 2 encounters before the stimulations and 2 after (follow-up). CE: cortical excitability; D1–D13: day (encounter) 1 to 13; IC: informed consent; NA: neuropsychological assessment; PainQ: pain questionnaires (*Douleur Neuropathique* 4 (DN4), McGill, and visual analogic scale (VAS)); rTMS: Repetitive Transcranial Magnetic Stimulation

Figure 1 shows the timeline of the study protocol.

### Subjects and recruitment

This study will include 36 patients from 18 to 60 years old, both genders, who have participated in previous observational prospective study of neuropsychological aspects and quality of life on victims of TBI. We will recruit patients who sustained DAI after TBI, based on clinical history and magnetic resonance imaging (MRI) findings confirming DAI. All eligible recruited patients will be randomly divided into two groups. During the screening period, all patients will receive a unique identification number, which will be used along the study to keep the staff blinded.

Throughout the study, regular documented clinical follow-up, evaluation instrument scales and possible telephone contact will be used to measure and maintain patient compliance.

#### Baseline measures

The following characteristics will be assessed at baseline: age, gender, handedness, time from TBI, level of schooling, mechanism of trauma, current use of medications, Glasgow coma scale at hospital admission, and prevalence of pain.

#### Evaluation of the prevalence of pain

The patients will be evaluated during the study about the presence of pain. If positive, this will be assessed with the visual analogic scale (VAS) score, DN4 (*Douleur Neuropathique* 4), and The McGill Pain Questionnaire. This questioning will be done before and after the rTMS sessions.

#### Randomization and blinding

Randomization will be done via a computer-produced randomized controlled table. Thirty-six patients will be randomized in 1:1 ratio into 2 groups: active coil group and sham coil group. This is a double-blind study, which the neuropsychologists and the patients (as well as their relatives) will be blinded for the group assignment. Only two of the authors involved directly with the rTMS application will assign participants to interventions.

#### Blinding and allocation concealment committee

This study involves the participation of a medical committee, not directly related to the patient group assignment, who can remove the blinding in caseany clinical condition arises that would be relevant to group assignment, adverse event, or patient dropout.

## Informed consent

Only patients who give informed written consent will be included in this study.

### Safety considerations and adverse events

This protocol is in accordance to the most recent guidelines about rTMS reported by Rossi et al. [31].

As per each meeting, patients will be questioned about adverse events and the information will be recorded and published. If a major adverse event occurs (e.g. seizure), the subject will receive medical assistance and further examination and investigation will be provided as needed. Family members will receive a feedback on the status of their relative who is enrolled in the study, and a contact number of the investigators and TMS clinic will be provided for further questions.

### Neuropsychological assessment

The neuropsychological evaluation will be performed in 3 phases, as follows:

- Evaluation 1: up to 3 months prior to rTMS sessions. The subjects will be submitted to the battery described below.
- Evaluation 2: up to 1 week after the completion of the rTMS sessions. The subjects will be submitted to the same battery as in Evaluation 1. This assessment aims to verify the rTMS’s early effects.
- Evaluation 3: 90 days after the conclusion of the rTMS sessions. This assessment will evaluate the delayed effects of the rTMS sessions.

**Fig. 2:**
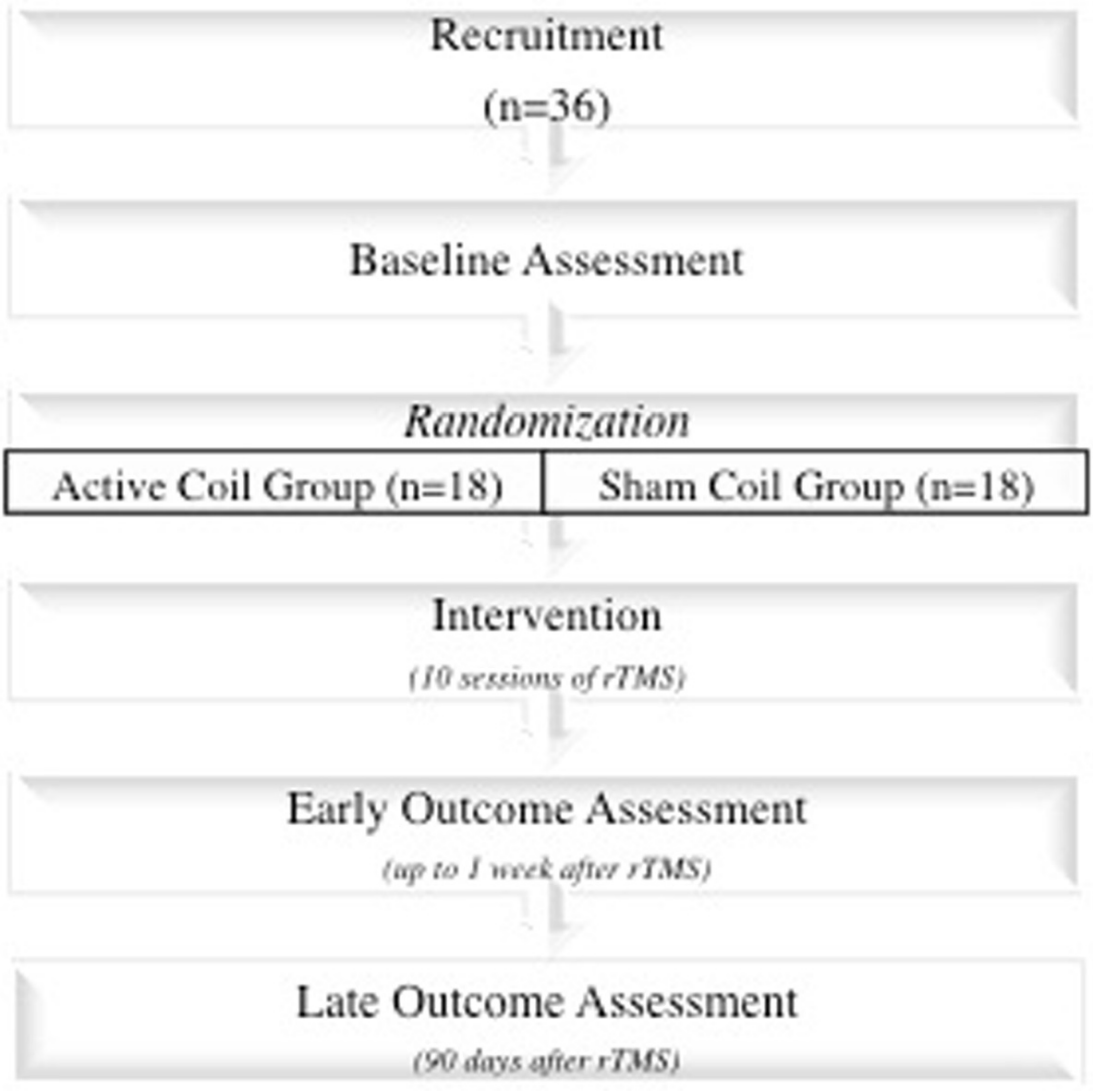
Study flow diagram: this study will recruit 36 patients from Hospital das Clínicas, University of São Paulo, Brazil. After baseline data collection, participants will be randomized to active coil group or sham coil group in a 1:1 ratio. Baseline assessment include standard history and clinical assessment, neurocognitive evaluation, prevalence of pain symptoms (assessed with the VAS, DN4, and The McGill Pain Questionnaire), MRI scan, and cortical excitability assessment with TMS. After the end of the intervention (rTMS), participants will undergo two revaluations (early and late data collection). DN4,*Douleur Neuropathique* 4, MRI, magnetic resonance imaging, TMS, Transcranial Magnetic Stimulation, rTMS, repetitive TMS, VAS, visual analogic scale

Figure 2 demonstrates the study protocol flow and highlights the three time points when the neuropsychological assessments will be held.

### Instruments

The following instruments will be applied during the three aforementioned evaluations: Beck Depression Inventory – BDI-II [32, 33]; State-Trait Anxiety Inventory (STAI) [34]; Focused and alternating attention: Trail Making Test parts A and B (TMTA and TMTB) [35]; Selective attention and inhibition: Stroop Test – Victoria version [36]; Verbal fluency [35, 36]; Five Points Test [37]; Symbol Digit Test (processing speed) [38]; Verbal learning and episodic long-term memory: Hopkins Verbal Learning Test – HVLT [39]; Brief Visuospatial Memory Test – BVMT [40]; Working memory (subscale of Wechsler Memory Scale – WAIS) [41]; Motor dexterity – Grooved Pegboard Test [42].

### Ethics committee and regulatory approval

The trial will be conducted in accordance with the ethical principles outlined in the Declaration of Helsinki, 1996. This research was approved by the Hospital das Clínicas, University of São Paulo Ethical Institutional Review Board (IRB #193.985/13). The present study has been approved by COSEPE (Sectorial Commission of Ethics in Research – Division of Psychology, Hospital das Clínicas – University of São Paulo) #18/2010 and CAPPESQ (Ethics Committee for review of research projects, Hospital das Clínicas – University of São Paulo) process #0097/11.

### Data analysis

The SPSS 19.0 software package for Windows (SPSS Inc., Chicago, IL, USA) will be used for all statistical analyses. The *T* test will be used for comparison between means and Chi-squared test for proportions. Non-parametric analysis will include Wilcoxon and Mann–Whitney tests. A significance level of *p* < 0.05 will be chosen for all tests. A fully specified statistical analysis plan will be written before unmasking the study in order to guarantee replicability and to avoid outcome selective reporting. All the analysis will be based on “as randomized” patients.

### Sample size

We have considered a difference of 1 standard deviation in TMTB, an 80 % power, and an alpha of 5 %, a minimum of 15 participants are needed for each group. We will add three patients per group to compensate for possible loss of follow-up. Thereafter, 18 patients will be allocated to each group.

## Discussion

It is a truism that TBI has a devastating global impact in terms of public health. Unfortunately, most of the cognitive rehabilitation strategies to date have shown only modest results. A multidisciplinary approach seems to be the most feasible intervention. Therefore, rTMS, as a non-invasive brain stimulation technique, has a particularly important appeal: the potential as a therapeutic instrument while obtaining insights into questions of brain function and pathophysiology of disabling cognitive processes. The results obtained from this study will be valuable in order to design larger randomized clinical trials and establish new concepts to the treatment of cognitive sequelae in patients with DAI.

## Trial status

This trial was registered on the website clinicaltrials.gov with the registration number NCT02167971. At the writing of this paper, we have already performed rTMS on nine patients and we are still recruiting subjects. The study began in January 2014 and the programmed completion date for the primary outcome is September 2015.

## Abbreviations

BDI-II: Beck Depression Inventory
BVMT: Brief Visuospatial Memory Test
CONSORT: Consolidated Standards of Reporting Trials
DAI: diffuse axonal injury
DN4: *Douleur Neuropathique* 4
DLPFC: dorsolateral prefrontal cortex
HVLT: Hopkins Verbal Learning Test
ICF: intracortical facilitation
ISI: interstimulus interval
MRI: magnetic resonance imaging
RMT: resting motor threshold
rTMS: Repetitive Transcranial Magnetic Stimulation
SICI: short-interval cortical inhibition
SPIRIT: Standard Protocol Items: Recommendations for Interventional Trials
STAI: State-Trait Anxiety Inventory
TBI: traumatic brain injury
TMS: Transcranial Magnetic Stimulation
TMTA: Trail Making Test part A
TMTB: Trail Making Test part B
VAS: visual analogic scale
WAIS: working memory (subscale of Wechsler Memory Scale)
